# Prognostic Significance of Hyponatremia in ST-elevation Myocardial Infarction/Heart Failure Patients

**DOI:** 10.7759/cureus.5673

**Published:** 2019-09-16

**Authors:** Vraj Shah, Nusrat Jahan

**Affiliations:** 1 Cardiology, California Institute of Behavioral Neurosciences and Psychology, Fairfield, USA; 2 Internal Medicine, California Institute of Behavioral Neurosciences and Psychology, Fairfield, USA

**Keywords:** stemi, hyponatremia, heart failure

## Abstract

ST-elevation myocardial infarction (STEMI) and heart failure (HF) are common, big-budget, debilitating and expanding diseases. Cardiovascular diseases, especially STEMI and heart failure have been known to cause 17.3 million deaths worldwide annually. Hyponatremia, delineated as a serum sodium (sNa) concentration <135 mmol/l, is a frequently seen electrolyte disturbance in practice and the prevalence, clinical impact; the prognostic factor of low SNa in STEMI/heart failure patients vary widely. The aim of this review is to assess its existence and comparing survival difference between hypo and normonatremic patients.

A comprehensive review of the published articles was conducted using database PubMed. We found a total of over 1400 articles. The inclusion criteria used for this review were age >65 years, published within the last 10 years, written in English, performed on human subjects and of studies such as reviews and randomized controlled trials (RCTs), especially for heart failure MeSH words. By applying this inclusion criterion, we found out 40 relevant articles which included 26 cohort studies, four clinical trials, four review articles, and six RCTs. In the analysis of 7,06,899 patients with STEMI/heart failure, hyponatremia was significantly linked to causing all-cause mortality, both short and long term (hazard ratio [HR] as continuous variable: 1.06; 95% confidence interval [CI]: 1.01-1.11; P = 0.026; HR as categorical variable: 1.71; 95% CI: 1.06-2.75; P = 0.028). The rates of rehospitalization were also higher (odds ratio, 1.68; 95% confidence interval, 1.32-2.14) along with prolonged hospital stays as well as a greater cost burden as compared to patients with normal serum sodium. It was existent not only in patients with reduced ejection fraction (HFrEF) but also in subjects with preserved ejection fraction (HFpEF) (HR 1.40, 95% CI 1.12 to 1.75, P = 0.004). Rise of first follow-up and discharge sodium does seem to have positive linkage on survival as well (hazard ratio [HR] 0.429, 95% CI 0.191-0.960, P = 0.04).

Hyponatremia is the most frequently encountered electrolyte abnormality in clinical practice and has a poor prognosis in both STEMI and heart failure patients. It exacerbates both short and long term mortality, rehospitalization rates, as well as the average length of stay in the hospital. Although it is still a mystery whether hyponatremia is just a marker of iller patients or the core of poor prognosis in patients with STEMI and HF, one thing is certain: timely recognition of patients at risk for developing hyponatremia could help to commence early treatment.

## Introduction and background

According to McMurray et al., ST-elevation myocardial infarction (STEMI) and heart failure (HF) are common, big-budget, debilitating, and expanding diseases [1]. Cardiovascular diseases, especially STEMI and heart failure have been known to cause 17.3 million deaths worldwide annually [2-4]. Hyponatremia, which refers to a serum sodium (sNa) concentration <135 mmol/l, is a common electrolyte disturbance that is seen frequently in clinical practice. Moreover, previous studies have shown the prevalence of hyponatremia in patients suffering from STEMI ranges between 12.5%-23.2% [5] and close to 25% in patients with HF [6].

Several mechanisms can explain the occurrence of hyponatremia in these patients, such as neurohormonal activation of the renin-angiotensin-aldosterone system (RAAS) as well as sympathetic overstimulation due to reduced stroke volume and subsequent underfilling of arteries [7-9]. Several observational studies and clinical trials have been carried out to appraise the prognostic impact of serum sodium levels at admission and during hospitalization of STEMI and HF patients. Accordingly, a strong alliance is established between the mortality of STEMI, HF patients and low sNa status at admission. Although it is still a mystery whether hyponatremia is just a marker of more ill patients or the core factor of poor prognosis in patients with STEMI and HF, one thing is certain: timely recognition of patients at risk for developing hyponatremia could help to commence earlier treatment.

The aim of this review is to identify the prognostic significance of hyponatremia in STEMI and HF patients, heart failure with both preserved (HFpEF) and reduced ejection fraction (HFrEF), and whether the correction of serum sodium during hospitalization and discharge could improve both short and long term adverse outcomes such as all-cause mortality, cardiac-specific mortality, rehospitalization, length of hospital stay, diuretic resistance as well as signs and symptoms.

## Review

A comprehensive review of published literature in PubMed was conducted in order to study the prognostic significance of hyponatremia in STEMI and HF patients. Articles included were those relevant to the theme of hyponatremia, STEMI and HF. A search was conducted using database PubMed with MeSH keywords such as hyponatremia, STEMI, and heart failure and subheadings such as analysis and mortality which gave us 1,254, 847 and 8,339 articles respectively. The inclusion criteria used for this review were age >65 years, published within the last 10 years, written in English, conducted on human subjects and included studies of reviews and randomized controlled trials (RCT) for heart failure MeSH word particularly. Yet, the total came to over 1400 articles after applying inclusion criteria. Out of these, there was a repetition involving 18 articles. A total of 40 pieces of literature were appropriate for this analysis. Out of these, 30 were included using their abstracts while 10 were included using full review. Among them, 26 were identified themselves as cohort studies (including 19 identified as a retrospective cohort and seven identified as a prospective cohort) [5, 10-34], four were review articles [35-38], four were clinical trials [39-42], and six identified themselves as RCTs [43-48]. A total of 70,6899 subjects comprise the study population. Table 1 shows the total number of articles obtained after applying inclusion criteria for MeSH keywords in PubMed. Table 2 shows appropriate articles for the literature review with the total number of study subjects.

**Table 1:** Keywords search after applying inclusion criteria in PubMed

Articles	Results	Results	Results
MeSH words	Hyponatremia	STEMI	Heart failure
Total articles	1254	847	8339
Year of publication (<10 years)	615	847	4089
Species (humans)	599	843	3960
Language (English)	565	827	3733
Age (>65)	297	601	2445
Study type (review, RCT)	-	-	509
Final articles	297	601	509

**Table 2:** Articles appropriate for the literature review, and the Total number study subjects.

Articles appropriate for the literature review	40
Articles included using abstract	30
Articles included using full text	10
Total number of study subjects	7,06,899

Based on study results, hyponatremia was significantly associated with short-term as well as long-term mortality in these patients. Rates of rehospitalization were also on a higher side as compared to patients with normal serum sodium. The average length of hospital stay and cost burden was much more. These findings were not only accurate for patients with preserved ejection fraction but also patients with reduced ejection fraction [5, 10-48].

In this analysis of 706,899 patients, we examined the interconnection between serum sodium and prognosis in STEMI and HF patients. A significant association was noted between serum sodium and outcome. As compared to normonatremia, hyponatremia was remarkably associated with poor prognosis in these study subjects.

In most studies, hyponatremia was found to be a standalone predictor of death in HF patients. In the current literature review, there was an immense and independent association between hyponatremia and term mortality (both short and long term, cardiac-specific and all-cause). The average length of hospital stay was prolonged in hyponatremic subjects than patients with normal sNa levels. Because it is a poor prognostic indicator and measure of the quality of life and cost, rehospitalization is usually included either alone or belonging to a composite clinical endpoint in studies examining various aspects of STEMI and HF. Based on study results, patients with low sNa levels had higher rehospitalization rates than normonatremic patients in our analysis. STEMI and HF patients with persistent low serum sodium, i.e., present both on admission and at discharge, have the highest mortality and rehospitalization rates compared with all-time normonatremia and the rise of first follow-up and discharge of sodium are positively correlated. These differences were significant in this study. This is concordant with previously reported studies emphasizing this same problem. It was evident even in patients with preserved ejection fraction. Table 3 shows the analysis of various studies such as cohort, RCT, and review.

**Table 3:** Analysis involving prospective and retrospective cohort studies, reviews and randomized controlled trials HFrEF - heart failure with reduced ejection fraction; HFpEF - heart failure with preserved ejection fraction; HR - hazard ratio; HF - heart failure; STEMI - ST-elevation myocardial infarction; IN-CHF - Italian network on congestive heart failure; ACE - angiotensin-converting enzyme; CI - confidence interval; NT-pro BNP - N terminal pro brain natriuretic peptide; sNa - serum sodium; MBDS - minimum basic data set; OR - odds ratio; RCT - randomized controlled trial; BNP - brain natriuretic peptide; KCCQ - Kansas city cardiomyopathy questionnaire

Author name/YOP	Study design	Sample size	Main points
Rusinaru et al., 2012 [37]	Systematic review	11301	Hyponatraemia has been negatively linked to heart failure patients with HFrEF. The connection between serum sodium and prognosis is unclear in heart failure with preserved (≥ 50%) ejection fraction (HFpEF). BY analyzing 14,766 patients from 22 studies, hyponatremia was solely predictive of demise in both HFrEF [adjusted hazard ratio (HR) 1.69] and HFpEF (adjusted HR 1.40, 95%, P for interaction 0.20).
Bavishi et al., 2014 [21]	Prospective cohort study	8862	Hyponatremia happens to be negatively linked in hospitalized patients with reduced ejection fraction. Still, there is insufficient information in roaming subjects with HF with preserved ejection fraction. By evaluating the prevalence, risk factors, and long-term outcomes of hyponatremia in ambulatory HFpEF and HFrEF in a cohort of 8,862 veterans. Hyponatremia is present at a similar frequency of over 10% in ambulatory patients with HFpEF and HFrEF. Hyponatremia is a sole prognostic marker of mortality across the spectrum of patients with HFpEF and HFrEF. In contrast, it is an independent predictor for hospitalization in patients with HFrEF along with HFpEF.
Abebe et al., 2018 [9]	Retrospective cohort study	388	Hyponatremia is shown to be negatively linked in STEMI and HF patients. It is significantly associated with death in advanced age. Hyponatremia is one of the major elements in the clinical outcome of heart failure subjects.
Hiki et al., 2018 [10]	Retrospective cohort study	584	Hyponatremia carries a poor prognosis in HF patients. There is a paucity of data regarding the value. This analysis showed increased rates of rehospitalization as well as mortality(both short-term and long-term) even with low-normal sodium levels.
Qureshi et al., 2013 [23]	Retrospective cohort study	11562	Hyponatremia has a strong positive linkage in patients with HF. However, insufficient data is there regarding its significance in patients presenting with STEMI. After retrospectively analyzing 11,562 subjects, our analysis showed that corrected and persistent hyponatremia in patients presenting with STEMI has been negatively linked to all-cause mortality both short-term and long-term and higher rates of rehospitalization. In certain subjects, timely correction of hyponatremia may ameliorate the survival of the patients.
Baldasseroni et al., 2011 [27]	Retrospective cohort study	4670	It has been proven that low serum sodium carries worse prognosis in HF and STEMI subjects. We analyzed 4670 patients signed up in the IN-CHF registry. Mild-to-moderate and severe hyponatremia (groups two and three) solely predicted first-year death. The connection between sodium concentration and demise was not linear and a drop of 1 mEq/l of sodium exacerbated death rate only for merits of sodium 142.9 mEq/l or less. This relationship was not modified by beta-blocker and ACE inhibitor therapies.
Mohammed et al., 2010 [31]	Retrospective cohort study	628	A total of 628 subjects who came to the emergency department with acutely decompensated heart failure were studied who were hospitalized. In multivariate Cox proportional hazards analysis showed hyponatremia to be an independent predictor of first-year demise (hazards ratio=1.72; 95% CI=1.22 to 2.37; P=0.001) as was an NT-proBNP concentration above the median value of 4690 pg/mL (hazards ratio=1.49; 95% CI=1.10 to 2.00; P=0.009). Subjects with low SNa and severely elevated NT-proBNP were much likely to evolve worsening kidney function during their course of hospital stay and had the highest first-year demise rates.
Méndez-Bailón et al., 2015 [18]	Retrospective cohort study	504860	This analysis assessed the incidence, average length of stay, comorbidities, rates of readmissions and deaths caused by hyponatremia in admissions for acute heart failure from the MBDS. A total of 5,04,860 patients with acute heart failure were enrolled, of whom 11,095 (2.2%) appeared with sNa.Overall deaths due to any cause in subjects with hyponatremia were 17% (1937 patients) versus 11% in normonatremic patients (53,820 patients). The probability of rehospitalization for patients with hyponatremia was 22% versus 17% in normonatremic group. Hyponatremia was negatively linked with mortality during hospitalization for acute heart failure with an odds ratio (OR) of 1.58, 95% CI, 1.50-1.66 (p<0.05).
Colin-Ramirez et al., 2015 [46]	RCT	38	Patients with HF (New York Heart Association classes II-III) were allocated to low or moderate-sodium consisting diet. Dietary intake was monitored using strict recording by the appropriate personnel. At six months, a post-hoc analysis using the dietary sodium uptake achieved (either above or below 1500 mg/dl) in all patients displayed a positive linkage between sodium intake ≤ 1.5 a day and enhancement in BNP and KCCQ scores. A dietary intervention reducing sodium intake was advisable, and the achievement of this sodium goal was associated with lower BNP levels and better quality of life in subjects with HF.

All randomized clinical trials, retrospective, and prospective cohort studies, as well as reviews, reveal a strong association between serum sodium and prognosis. Collectively, these data do provide sufficient evidence that low sNa carries worse prognosis in these patients.

Various mechanisms contribute to low serum sodium in these patients such as low stroke volume, low cardiac output with subsequent underfilling of arterioles and activation of RAAS, the non-osmotic release of vasopressin and severe sympathetic stimulation causing water retention, vasoconstriction causing dilutional hyponatremia. The level of serum sodium may affect the transmembrane potentials in heart cells, the formation of regulatory proteins, enzymes and muscle excitation.

The current literature review did not include STEMI and HF patients below 65 years with hyponatremia, hyponatremia associated with diabetes, advanced age, increase blood urea nitrogen and diuretics. After adjusting for these variables, the results could have been altered. However, one thing is certain that the dynamic monitoring of serum sodium could help physicians identify these high-risk patients and stratify risk for optimal management.

## Conclusions

Hyponatremia is the most frequently encountered electrolyte abnormality in clinical practice carries poor prognosis in both STEMI and HF patients. It exacerbates both short- and long-term mortality, rehospitalization rates, the average length of stay in the hospital. The rise of first follow-up sodium during hospitalization as well as during discharge is linked with better outcomes. It is prevalent not only in patients with reduced ejection fraction but also in preserved ejection fraction. Although it is still a mystery whether hyponatremia is just a marker of more ill patients or core of the problem, intense monitoring of sNa could help physicians and researchers optimizing appropriate treatment regimen and to stratify risk management.
